# An Improved Approach for Terrain Correction: Application to Northeast Asia’s Highest Peak (Mt. Jade, Taiwan)

**DOI:** 10.3390/s90906604

**Published:** 2009-08-24

**Authors:** Kwo-Hwa Chen

**Affiliations:** Department of Real Estate and Built Environment, National Taipei University / No. 151, University Road, San Shia, Taipei 23741, Taiwan; E-Mail: khchen@mail.ntpu.edu.tw; Tel.: +886-2-2674-8189 ext. 67421; Fax: +886-2-8671-5308

**Keywords:** terrain correction, cone-section, cylinder prism, Gaussian quadrature

## Abstract

Mt. Jade (or “Yushan” in Chinese) is the highest peak in northeast Asia. The topography is very rugged and complicated. Such terrain makes it difficult to obtain the correct results for terrain corrections (TCs). This paper developed an improved approach, named cone-section method, to compute the TCs of the Mt. Jade area using a high-resolution digital elevation model (DEM) on a 9″ × 9″ grid. The corrections were calculated to the distance of 100 km with an average rock density of 2.57 × 10^3^ kg·m^−3^. This investigation compared the results of TCs from the cone-section method with those from the cylinder prism and Gaussian quadrature methods using a 9″ × 9″ elevation grid for the inner zone and a 90″ × 90″ elevation grid for the outer zone. The inner and outer radii were set to 20 and 200 km, respectively. The comparisons showed that the cone-section algorithm is consistent with the Gaussian quadrature. Furthermore, the cone-section method is an appropriate approach for TCs in high elevation areas, yielding results that outperform the cylinder prism method.

## 1. Introduction

Various geodesy applications require terrain corrections (TCs), for example, geoid estimation [1], orthometric correction [2], and the interpretation of crustal structure [3]. Previous studies used many methods for TC estimations, such as fan-shaped prism [4,5], cylinder prism [6], FFT [7–9], and Gaussian quadrature [10,11]. Among all these methods, researchers theoretically regard the Gaussian quadrature formula as the most precise method because it yields more improved and high-frequency variations in TCs than do the other algorithms [11]. Generally, the discrepancies of TCs between the cylinder prism and Gaussian quadrature methods are smaller than differences between the FFT and Gaussian quadrature methods. For the most part, the cylinder prism and FFT methods may still have room for improvement to calculate the effect of terrain in high elevation areas [11]. This paper develops an improved approach, based on the cone-section method, to estimate the TCs in high relief regions like Mt. Jade, with an elevation of 3,951.798 m [12]. The proposed method uses a high resolution digital elevation model (DEM) with a resolution of 9″ × 9″ grid horizontally and an average rock density of 2.57 × 10^3^ kg·m^−3^ [5]. The aim of this paper is to demonstrate the algorithms of this cone-section method and to make comparisons among TCs from the cone-section, Gaussian quadrature, and cylinder prism methods.

## 2. The Cone-section Method

**Figure 1.**
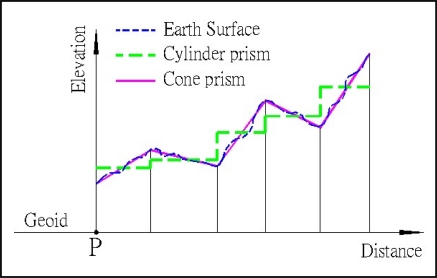
Relationships among earth surface, cylinder prism and cone prism.

**Figure 2.**
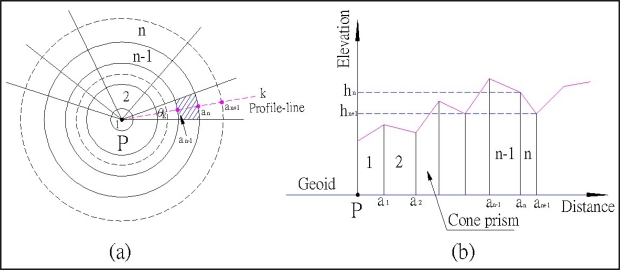
Geometry of cone-section method.

As shown in Figure 1, the cone-section method uses cone prisms to fit the earth surface. In Figure 2b, the cone-section method uses the inner and outer elevations of the two intersection points (e.g., the elevations of the points of a_n−1_ and a_n_) derived from DEM to compute the attraction of each cone prism. Obviously, the cone-section method is better than the cylinder prism method that uses the average elevation from the inner and outer elevations of each cylinder prism.

The cone-section method yields TCs using the following steps: 1) equally divide the terrain surrounding the surveying site into several small cone prisms (Figure 2a); 2) plot the inner and outer elevations of two intersection points (e.g., the elevations of the points of a_n−1_ and a_n_) of each cone prism from a high resolution DEM with a 9″ × 9″ grid; 3) estimate the attraction of each cone prism; 4) obtain the total TCs by summing the contributions from all such cone prisms as Figure 2 shows. Figure 2a shows the realistic depictions of topography variations increase with the number of divided sectors.

**Figure 3.**
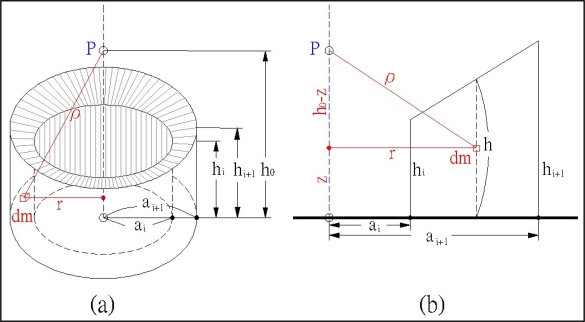
Attraction of each cone prism in cone-section method.

As Figure 3 demonstrates, the vertical component of the attraction of a differential mass may be expressed at point P as:

(1) $${\mathrm{dF}}_{z}=G\frac{\mathrm{dm}}{\rho^{2}}\cdot \frac{z-h_{0}}{\rho }$$

where:

*G*: the gravitational constant
*dm*: the differential mass
*h*_0_: the elevation of surveying site P
*ρ*: the distance between surveying site P and differential mass dm as shown in Equation (2) and Figure 3b

(2) $$\rho =\sqrt{r^{2}+{\left(z-h_{0}\right)}^{2}}$$

where:

*r*: the horizontal distance between surveying site P and differential mass dm
*z*: the elevation of differential mass dm

The integral form of Equation (1) is

(3) FZ=Gδ∫θ=02π∫aiai+1∫0h(z−h0)⋅dz⋅r⋅dr⋅dθ(r2+z2)32

where:

*δ*: the average rock density (set to 2.57×10^3^ kg·m^−3^ [5])
*a_i_*: the inner radii
*h_i_*: the inner elevation
*a*_*i*+1_: the outer radii
*h*_*i*+1_: the outer elevation

Equation (3) could be transformed to:

(4) FZ=Gδ∫θ=02π∫aiai+1∫−h0sr+k−h0z′⋅dz′⋅r⋅dr⋅dθ(r2+z′2)32=−2πGδ∫aiai+1[(s2+1)⋅[(r+s(k−h0)s2+1)2+(k−h0)2(s2+1)2]]−12⋅r.dr+2πGδ(ai+12+h02−ai2+h02)

where:

(5) $$z\prime =z-h_{0}$$

(6) dz′=dz

(7) $$s=\frac{h_{i+1}-h_{i}}{a_{i+1}-a_{i}}$$

(8) $$k=\frac{a_{i+1}h_{i}-a_{i}h_{i+1}}{a_{i+1}-a_{i}}$$

and:

*s*: the slope parameter of Equation (9)
*k*: the intercept parameter of Equation (9)

where:

(9) h=sr+k

The solution form of Equation (4) is:

(10) FZ=−2πGδ(s2+1)∫ai+pai+1+p(u2+q2)−12⋅(u−p)⋅du+2πGδ(ai+12+h02−ai2+h02)=−2πGδ(s2+1)[((ai+1+p)2+q2−(ai+p)2+q2)−p⋅ln(ai+1+p)2+q2+ai+1+p(ai+p)2+q2+ai+p]           +2πGδ(ai+12+h02−ai2+h02)

where:

(11) $$p=\frac{s\left(k-h_{0}\right)}{s^{2}+1}$$

(12) $$q^{2}=\frac{{\left(k-h_{0}\right)}^{2}}{{\left(s^{2}+1\right)}^{2}}$$

(13) u=r+p

Assuming the attraction value is positive in a downward direction, the attraction of each cone-section prism (Figure 2) is:

(14) $$\begin{array}{ll} {\left(F_{Z}\right)}_{k} & =F_{Z}\times \frac{\theta_{k}}{2\pi }\\ & =\frac{\theta_{k}G\delta }{\sqrt{\left(s^{2}+1\right)}}\left[\left(\sqrt{{\left(a_{i+1}+p\right)}^{2}+q^{2}}-\sqrt{{\left(a_{i}+p\right)}^{2}+q^{2}}\right)-p\cdot \text{ln}\frac{\sqrt{{\left(a_{i+1}+p\right)}^{2}+q^{2}}+a_{i+1}+p}{\sqrt{{\left(a_{i}+p\right)}^{2}+q^{2}}+a_{i}+p}\right]\\ & -\theta_{k}G\delta \left(\sqrt{{a_{i+1}}^{2}+h_{0}^{2}}-\sqrt{a_{i}^{2}+h_{0}^{2}}\right) \end{array}$$

Divide the topography surrounding the surveying site P into n concentric circles with k equal cone prisms (Figure 2), then obtain the total TCs of P by summing the contributions from all cone prisms as:

(15) $${\left(\mathrm{TCs}\right)}_{p}=\sum_{i=1}^{n}\sum_{j=1}^{k}{\left(F_{Z}\right)}_{\mathrm{ij}}$$

## 3. Results and Discussion

**Figure 4.**
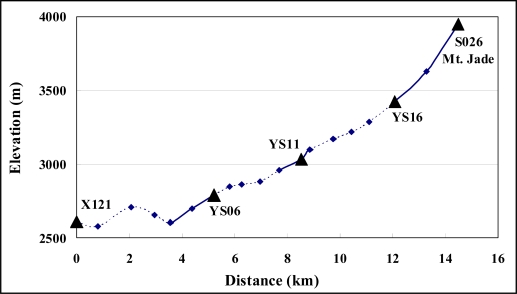
Surveying sites of Mt. Jade area.

Mt. Jade is the highest peak in northeast Asia. The topography of the area is very rugged. Relative gravity measurements of the Mt. Jade area using a LaCoste and Romberg type G gravimeter were obtained in [12]. The absolute gravity values in the region range from 978,280 mgal (on X121, which is the first-order benchmark of Taiwan) to 977,954 mgal (on S026, a surveying monument at Mt. Jade peak). This study used a high-resolution digital elevation model (DEM) for TC computations from five surveying monuments (Figure 4). The grids of the DEM were generated by [2] using a total of 6,421,075 points of elevation data in Taiwan, covering the area over 21.5°–25.5°N and 119.5°–122.5°E. This paper used this DEM on a 9″ × 9″ grid. The cone-section method corrections were calculated to the distance of 100 km with an average rock density of 2.57 × 10^3^ kg·m^−3^ [5]. The investigation also estimated the TCs using the Gaussian quadrature [11] and the cylinder prism methods [10] for comparing the results with cone-section method results. The procedure split the topography surrounding the surveying site into two parts because computations by the Gaussian quadrature method for TCs are relatively time consuming compared to the cone-section and cylinder prism methods. The first part, the inner zone, had a fine elevation grid (on a 9″ × 9″ grid) and the second part, the outer zone, had a coarse elevation grid (on a 90″ × 90″ grid). [10] recommended such a strategy. Based on this strategy, [11] developed a program “tcq.f” in FORTRAN 90 for implementing the Gaussian quadrature method. This paper set the inner and outer radii for the determinations of Gaussian quadrature to 20 and 200 km (recommended by [11]). Furthermore, this paper utilized a program “tc.f” (in FORTRAN 90, developed by [10]) for the cylinder prism method. Program “tc.f” also divided TC computations into an inner zone and an outer zone, but “tc.f” did not take into account the innermost zone effect as the Gaussian quadrature method does. The proposed method first estimated TCs on the same 9″ × 9″ grid as the elevation grid, and then determined the TCs from the surveying monuments of Mt. Jade area by interpolations using the Newton-Gregory polynomial [10]. These cylinder prism method corrections were calculated to the distance of 200 km, which was longer than the cone-section method which uses 100 km.

Table 1 shows the TC results from the cone-section, the Gaussian quadrature, and the cylinder prism methods. Table 2 shows the comparisons among TCs from these three methods. As [11] points out, the Gaussian quadrature method picks up more high-frequency variations in TCs than does the cylinder prism method. In Table 2, the differences of TCs between the Gaussian quadrature method and the cylinder prism method range from +2.837 to +8.235 mgal, except for surveying site S026 on Mt. Jade peak. The differences show that the cylinder prism method may still have room for improvement. In contrast to the results of the cylinder prism method, the cone-section method obtained smaller TC differences than the cylinder prism method did, ranging from +0.832 to +2.320 mgal except for the surveying site S026. TC results from the cone-section method were closer to results from the Gaussian quadrature method than the cylinder prism method. Table 3 shows the computation times from the cone-section, the Gaussian quadrature, and the cylinder prism methods. Obviously, the Gaussian quadrature method is slower than the cone-section and cylinder prism methods. Furthermore, the cone-section method is the fastest method among those three methods.

These comparison results demonstrate that the cone-section method is an appropriate approach for TCs in high elevation areas, yielding results that outperform the cylinder prism method. Researchers theoretically regard the Gaussian quadrature formula as the most precise method for estimating TCs [11]. However, the intention of the Gaussian quadrature method is point-by-point computations and would waste computing time if used for grid-wise computations. Both the cone-section and cylinder prism methods are quicker than the Gaussian quadrature method.

As Tables 1 and 2 show, the TCs from the S026 surveying site obtained by the cone-section and cylinder prism methods are significantly different from the Gaussian quadrature method TC, compared to the results obtained from the other sites (X121, YS06, YS11, and YS16). In order to analyze the near-zone effects, TCs from another five points located on five different peaks, respectively, were estimated by those three methods. The results are shown as Tables 4 and 5. There are no significant differences among results from the cone-section, the Gaussian quadrature, and the cylinder prism methods. The reason for this difference from Tables 1 and 2 could be that the topography variations surrounding Mt. Jade peak are still uncertainty. One of the best ways to improve the estimation results of TCs for Mt. Jade peak is to get finer DEM grids, specially surrounding the Mt. Jade peak, than those used in this paper. In addition, Tables 4 and 5 also show that the cone-section method is an appropriate approach for TCs in comparison with the cylinder prism method.

## 4. Conclusions

This paper used the cone-section method to compute the TCs for five surveying sites from the Mt. Jade area. This method obtained a significant improvement in TC determination. The cone-section method yielded better TCs than did the cylinder prism method. TC computations only used the information of topography surrounding the surveying sites from 0 to 100 km in the cone-section method. The cylinder prism and Gaussian quadrature methods corrections were calculated to the distance of 200 km. Although the Gaussian quadrature method obtained more high-frequency variations in TCs than do the other two methods, it required more computer time than the above two methods. The results in this paper suggest the cone-section method is an appropriate approach for TCs in high elevation areas. The cone-section method yields results that outperform the cylinder prism method and saves computation time over the Gaussian quadrature method. However, concerns about the precision of topography surrounding the surveying sites, namely Mt. Jade peak S026, limit current study conclusions. In addition, determining the reason for the significant differences in the estimations of TCs on Mt. Jade peak (site ID: S026) remains for finer accuracy of digital elevation model (DEM) than those used in this paper.

## Figures and Tables

**Table 1.** TCs (in mgal) from five surveying sites of Mt. Jade area using different methods.

**Site ID**	**Cone-section**	**Gaussian quadrature**	**Cylinder prism**	**Longitude (degree)**	**Latitude (degree)**	**Elevation (m)**
**X121**	25.509	24.251	27.088	120.890	23.487	2,610
**YS06**	36.049	35.217	40.583	120.910	23.472	2,792
**YS11**	42.983	41.661	49.896	120.931	23.464	3,036
**YS16**	45.006	42.686	46.132	120.949	23.467	3,426
**S026**	90.660	114.387	90.651	120.957	23.470	3,952

**Table 2.** Differences (in mgal) in TCs from different methods.

**Site ID**	**Between Cone-section & Gaussian quadrature**	**Between Cylinder prism & Gaussian quadrature**
**X121**	1.258	2.837
**YS06**	0.832	5.366
**YS11**	1.322	8.235
**YS16**	2.320	3.446
**S026**	−23.727	−23.736

**Table 3.** Computation times in TCs from different methods.

**Method**	**Number of calculated points**			
	**5**	**100**	**500**	**1,000**

	**Computation time (second)**			
**Gaussian quadrature**	1.17	7.04	31.85	62.65
**Cylinder prism**	1.38	4.52	11.21	21.96
**Cone-section**	1.41	3.97	9.34	17.75

**Table 4.** TCs (in mgal) from five surveying sites located on different peaks using different methods.

**Site ID**	**Cone-section**	**Gaussian quadrature**	**Cylinder prism**	**Longitude (degree)**	**Latitude (degree)**	**Elevation (m)**
**M028**	29.564	28.553	30.607	121.143	23.752	2,515
**M477**	26.181	25.753	29.108	121.317	24.187	2,817
**E019**	67.942	66.375	68.443	121.002	22.977	2,930
**S048**	116.686	115.724	120.204	120.761	22.627	3,090
**M089**	37.496	36.704	36.591	121.285	24.152	3,236

**Table 5.** Differences (in mgal) in TCs from different methods.

**Site ID**	**Between Cone-section & Gaussian quadrature**	**Between Cylinder prism & Gaussian quadrature**
**M028**	1.011	2.054
**M477**	0.428	3.355
**E019**	1.567	2.068
**S048**	0.962	4.480
**M089**	0.792	−0.113
